# How Health Insurance Instability Differentially Impedes Access to Sexual and Reproductive Healthcare, by Race/Ethnicity and Nativity

**DOI:** 10.1111/1475-6773.70049

**Published:** 2025-10-07

**Authors:** Hannah Olson, Ayana Douglas‐Hall, Madeleine Haas, Megan L. Kavanaugh

**Affiliations:** ^1^ Guttmacher Institute New York USA; ^2^ Hunter College City University of new York New York USA

**Keywords:** contraceptive access, insurance churn, insurance instability, nativity, race/ethnicity, sexual and reproductive healthcare, uninsurance

## Abstract

**Objective:**

To document differential risk of insurance instability by race/ethnicity and nativity and investigate the effect of insurance instability on subsequent sexual and reproductive health care utilization and contraceptive access.

**Study Setting and Design:**

We draw on data from the Surveys of Women (SoW), longitudinal household surveys conducted by NORC at the University of Chicago in Arizona, Iowa, New Jersey, and Wisconsin, weighted to reflect the population of women aged 18–44 in each state. SoW respondents included in this analysis were interviewed 2–4 times between 2018 and 2022 about their sexual and reproductive health‐related experiences. We use race‐stratified population averaged logistic regressions to model the risk of insurance churn and insurance loss for US‐born vs. foreign‐born people with the capacity for pregnancy, by race/ethnicity. Then, we use within‐between (hybrid) logistic regressions to model the effect of insurance instability on subsequent sexual and reproductive health care utilization and contraceptive access outcomes, including receipt of any sexual and reproductive health care, receipt of contraceptive care, experiencing barriers to obtaining contraception, and contraceptive use.

**Data Sources and Analytic Sample:**

Our analytic sample includes 12,208 observations from 4558 respondents between the ages of 18 and 44 who were assumed to have the capacity for pregnancy. Respondents were maintained in the sample if they were neither pregnant nor infertile and had non‐missing information on key variables.

**Principal Findings:**

Insurance loss was much more common among foreign‐born compared to US‐born people, particularly those who were racially or ethnically minoritized, with foreign‐born BIPOC and foreign‐born Hispanic respondents experiencing insurance loss 2.5 and 3 times as often as their US‐born counterparts, respectively. Meanwhile, findings from our hybrid models suggest that losing insurance was associated with a five percentage point reduction in the probability of subsequent utilization of sexual and reproductive health care (∆*p* = −0.046, *p* < 0.05, SE = −0.02) and a five percentage point increase in the probability of experiencing subsequent barriers to obtaining preferred contraception (∆*p* = 0.053, *p* < 0.001, SE = 0.01).

**Conclusion:**

The disproportionate burden of insurance instability among immigrant people of color may exacerbate barriers to sexual and reproductive health care and contraceptive access for a population that already experiences high barriers to obtaining this care relative to non‐Hispanic White people.

**Summary**

- What is known on this topic?
  ○ Health insurance coverage is crucial for protecting access to needed healthcare and promoting overall and sexual and reproductive health well‐being in the United States.
  ○ Insurance instability is common in the US and the burden of uninsurance is disproportionately borne by the most marginalized in society, including immigrants and people of color.
  ○ Insurance instability has been linked to disruptions in care, particularly during the perinatal period.
- What this study adds?
  ○ Insurance churn is common for most people, but insurance loss is much more common among foreign‐born vs. US‐born people of reproductive age, particularly those who are racially or ethnically minoritized.
  ○ While insurance churn without a lapse in coverage was not, insurance loss was strongly associated with lower sexual and reproductive health care utilization and more barriers to obtaining contraception.
  ○ The disproportionate burden of insurance instability among immigrant people of color may exacerbate barriers for a population that already experiences relatively high barriers to obtaining this care.

## 1 Introduction

Contraception and related sexual and reproductive health care plays an important role in facilitating overall and sexual and reproductive health and well‐being [1]. Continuous and reliable health insurance coverage is crucial both for protecting sexual and reproductive health care and contraceptive access and for promoting health throughout the life course [2, 3, 4, 5, 6]. People who have health insurance are more likely to use contraception [7, 8], especially preferred methods [9], and have lower contraceptive expenditures than the uninsured [10]. Additionally, concerns about cost and uninsurance lead some to postpone or forgo needed sexual and reproductive healthcare [11]. Uninsurance and insurance instability, meanwhile, are disproportionately borne by the most marginalized in society, threatening efforts toward sexual and reproductive health equity, defined as the ability for all people to “have what they need to attain their highest level of sexual and reproductive health.” [1].

Given the crucial role insurance plays in protecting overall and sexual and reproductive health, a growing body of research has demonstrated the importance of continuous coverage and the adverse outcomes associated with changing insurance [12] or experiencing even short gaps in coverage [13, 14, 15]—a combined set of outcomes we refer to as “insurance instability.” Unlike point‐in‐time comparisons by insurance status, research on insurance *instability* acknowledges that coverage can be volatile, particularly among people experiencing more frequent changes in the factors that shape eligibility for coverage, such as income, employment, or exposure to exclusionary public benefits policies and immigration enforcement [12, 16]. Experiencing more volatility in household income and more often occupying temporary and/or low‐wage jobs with no benefits also puts individuals at the lower end of the income distribution at disproportionate risk of insurance instability [12, 16, 17].

While uninsurance clearly hinders access to care, churning between plans may also disrupt access by changing what services or medications are covered, how and where they are obtained, and how much they cost under different plans [18]. While some evidence suggests this type of churn can disrupt access to care [12, 19], most existing research has focused on churn with resulting coverage gaps [13, 14, 15]. Thus, while even brief periods of uninsurance are likely to create barriers to healthcare, whether churning between plans without a gap in coverage may also be disruptive is less clear [12]. Existing research also suggests that insurance instability poses unique risks to ensuring consistent access to crucial sexual and reproductive health care, including avoiding undesired pregnancies, receiving adequate perinatal care, and the prevention and treatment of STIs or cancers [19, 20, 21]. Still, questions about the roles of insurance loss vs. churn as well as how insurance instability may be differentially felt by people who occupy one or more marginalized identities remain largely unanswered.

For immigrants in particular, safety net eligibility requirements can be complex and vary across states, creating confusion for immigrants and their families who need subsidized coverage [22, 23, 24]. Many immigrants who are eligible for public insurance coverage remain uninsured due to misunderstandings about requirements, difficulty navigating enrollment, or fears about immigration enforcement within mixed‐status households. In addition to representing a multitude of sociopolitical, economic, cultural, and linguistic backgrounds, immigrants also occupy a range of immigration statuses that present different barriers to accessing employment opportunities, insurance coverage, and other publicly supported resources [25]. While immigrants represent a diversity of experiences and backgrounds, many are racialized as “minorities” upon integrating into American society. This process of racialization leaves immigrants of color vulnerable not only to the political strains of occupying a potentially tenuous legal status but also the myriad ways in which structural racism assigns risks and rewards for people in the US along a racial hierarchy [26].

The current study uses longitudinal survey data to examine the role of insurance instability on sexual and reproductive health care utilization and contraceptive access. Continuous insurance coverage is a key pathway for ensuring access to needed healthcare [19, 20, 21] and highlighting where this pathway is disrupted and for whom is critical to revealing where systems need to be adjusted and rebuilt to ensure sexual and reproductive health equity.

This study extends our understanding of how insurance disruptions shape sexual and reproductive health equity in three ways. First, we use a longitudinal approach that is well‐suited to investigating potential causal links between insurance churn vs. insurance loss and utilization or access to sexual and reproductive health care. Previous longitudinal studies investigating the effects of insurance instability on sexual and reproductive health over time have focused either on gaps in coverage during the perinatal period [20, 27] or on the role of insurance continuity on contraceptive method choice [21]. There are few studies investigating the consequences of insurance churn separately from insurance loss [12, 28] and we are not aware of any studies that investigate how either type of insurance instability impacts prospective utilization of broader sexual and reproductive health care or contraceptive access. Second, while most of the existing research on the effects of insurance instability centers around childbirth [27, 29, 30], we look beyond the perinatal period to reveal the broader sexual and reproductive health consequences of insurance instability for all people of reproductive age. Lastly, we hypothesize that immigrants, especially immigrants of color, may be particularly vulnerable to disruptions in sexual and reproductive health services as policies regarding reproductive rights, healthcare, and immigration become entwined within hostile policy landscapes [31]. Evidence of a differential impact of insurance instability on healthcare utilization and access among immigrants and racially or ethnically minoritized people is scarce. Thus, we contribute to this body of work by highlighting the unique vulnerabilities to insurance instability and its consequences for people who occupy intersecting identities that have been historically marginalized in American society. To these ends, the current study has two primary research aims.

Our first research aim is to document the differential risk of insurance churn and loss of insurance between US and foreign‐born people with the capacity for pregnancy, stratified by racial/ethnic minoritization. Our second research aim is to assess the role of insurance instability in shaping subsequent sexual and reproductive healthcare utilization and contraceptive access.

## 2 Methods

### 2.1 Study Design and Sample

We draw on data from the Surveys of Women (SoW), longitudinal household surveys conducted by NORC at the University of Chicago across multiple states. For this analysis, we draw on SoW data from Iowa, Arizona, New Jersey, and Wisconsin, states that were originally identified under the Reproductive Health Impact Study [32], a larger multi‐year initiative to track the impacts of policy changes on publicly funded family planning healthcare systems and their patients. NORC fielded baseline surveys in these states between October 2019 and July 2020 and two rounds of follow‐up surveys in 2021 and 2022, gathering experiences of sexual and reproductive health and healthcare, including contraceptive use and preferences. All data from the SoW are weighted to reflect the states' population of reproductive‐aged women. Additional details regarding SoW data and methods are published elsewhere [33, 34]. NORC's Institutional Review Board approved the data collection protocols. This secondary data analysis utilizes only de‐identified data and was exempt from further review.

Our analysis included respondents who were neither pregnant nor infertile and had non‐missing information on our key outcome, exposure, and control variables. Notably, we dropped respondents who reported sterilization (e.g., tubal ligation, hysterectomy, oophorectomy) as their only method of contraception due to inconsistencies in questionnaires across states and time points. We include a PRISMA‐style case loss diagram of all sample exclusions in our Supporting Information. Our final analytic sample includes 12,208 observations from 4558 people with the capacity for pregnancy.

### 2.2 Measures

#### 2.2.1 Measures of Insurance Instability

Our analysis includes two measures of insurance instability: insurance churn and insurance loss. We define insurance churn as a change in insurance type with no gap in coverage. As churn can only be measured across two time points, no respondent was marked as having experienced insurance churn at baseline or any time point at which the last observation was more than 12 months prior.

We define insurance loss as any short‐term gap in insurance coverage or a shift from being insured at the preceding interview to being uninsured at follow‐up. At baseline, respondents are marked as having lost insurance in the prior year if they reported being uninsured at some point in the prior 12 months. Exact survey questions and further information on variable construction are provided in Supporting Information.

#### 2.2.2 Measures of Sexual and Reproductive Healthcare Utilization and Contraceptive Access

Key dependent variables within our second research aim include receiving any sexual and reproductive health care in the past 12 months, receiving contraceptive care in the past 12 months, experiencing any delay or trouble getting a preferred contraceptive method in the past 12 months, and current contraceptive method use. Our measure of contraceptive care includes receiving a birth control method or prescription, having a check‐up or medical test related to birth control, or receiving counseling on birth control methods. Receipt of any sexual and reproductive health care includes receiving any of these contraceptive services or counseling regarding pregnancy and fertility desires, a pregnancy test, or a general gynecological exam. Related survey questions and further information on variable construction are provided in Supporting Information.

#### 2.2.3 Demographic Measures

We collapse race/ethnicity into three categories: White non‐Hispanic, Hispanic, and other non‐Hispanic BIPOC—combining those identifying as Black, Asian or other Pacific Islander, American Indian or Alaskan Native, or multiracial. Small cell sizes prevented us from further disaggregating these other racial/ethnic groups. Nativity is a binary variable indicating whether the respondent was born in the United States.

Our analysis also controls for age, educational attainment at baseline, employment status, LGBTQ+ sexual orientation or gender identity at baseline, and marital status. Age is a three‐category variable distinguishing between older adults (26–34 and 35+) and younger adults (18–25). Educational attainment includes three categories: high school or less, some college or an Associate's degree, or a Bachelor's degree or higher and is treated as time‐invariant, taking each respondent's educational attainment at baseline. Employment is binary, with people who are unemployed or out of the workforce categorized as “not employed.” LGBTQ+ status is a binary variable identifying respondents who are either non‐cisgender or non‐heterosexual at baseline as LGBTQ+. Current marital status is binary and combines respondents who have never been married with those who were widowed or divorced as “not currently married.”

Lastly, as we have pooled data across states in our analyses, we also control for the state (New Jersey, Iowa, Wisconsin, or Arizona) and calendar year (2018–2022) in which the interview took place. While age, employment status, and marital status may vary within respondents across time, nativity, race/ethnicity, LGBTQ+ orientation/identity, and state of residence are treated as time‐invariant by taking baseline values for each respondent.

#### 2.2.4 Correlates of Demand for Sexual and Reproductive Healthcare

In the analysis for our second research aim, we include two additional correlates of sexual and reproductive health which represent two common but not comprehensive drivers of demand for contraception and sexual and reproductive health care. The first is a binary variable measuring whether the respondent had penile‐vaginal sex within the 3 months preceding the interview. The second is a continuous variable rating the respondents' desire to avoid pregnancy (DAP) using a psychometrically validated measure of a person's preferences about a future pregnancy and childbearing [35]. As per the DAP authors' recommendation, we use mean DAP scores in our analysis, resulting in ranges of 0 (low desire to avoid pregnancy) to 4 (high desire to avoid pregnancy).

### 2.3 Data Analysis

We first describe our baseline sample stratified by race/ethnicity and nativity, including weighted percentage distributions by state of residence, age, education, employment, LGBTQ+ status, marital status, insurance status, and recent penile‐vaginal sexual activity as well as mean DAP scores. We also provide weighted distributions across our key dependent variables at baseline.

To address our first research aim, we used race‐stratified weighted population averaged logistic regression models and Stata's xtlogit command to model relative odds of (1) experiencing insurance churn and (2) losing insurance coverage for those born within vs. outside the United States, within each of our three racial/ethnic subgroups. We used all available observations from respondents with at least two consecutive observations over the study period and accounted for repeated observations within respondents in our unbalanced panel. We also controlled for correlates of insurance access such as age, current marriage, current employment, and education as well as survey year and state of residence. We then used average marginal effects to calculate predicted probabilities for each outcome among each nativity and racial/ethnic subgroup. We graphically depict these probabilities in our results and provide the underlying regression output in Supporting Information.

To address our second research aim, we employed within‐between (hybrid) logistic regressions to model the effect of experiencing insurance instability on the odds of (1) receiving any sexual and reproductive health care, (2) receiving contraceptive care, (3) experiencing barriers to obtaining wanted contraception, and (4) use of contraception. We constructed our hybrid models using Stata's user‐written *xthybrid* command, which employs a multi‐level framework (e.g., *meglm* in Stata) and adds both the person‐specific mean of each variable and the deviation from that mean for time‐varying variables [36, 37]. We replicated our hybrid models using Stata's *meglm* program to allow for the transformation of our coefficients into marginal effects with Stata's *margins command*, which is not compatible with the user‐written *xthybrid* command. Using this hybrid approach allows us to differentiate between person‐level mean effects (i.e., between‐person differences) and the effects of deviations from that mean (i.e., within‐person differences) of our key time‐varying independent variables while also allowing for random effects estimations of control and other key variables that are time invariant (e.g., race/ethnicity and nativity). Between‐person coefficients represent average differences in outcomes *between* people who had and those who had not experienced insurance instability. The within‐person coefficient, on the other hand, reflects a comparison of outcomes between a person who has experienced one of our two measures of insurance instability to the outcomes of that *same* person at a time when they had not experienced that type of insurance instability. Like a fixed‐effects model, the within‐person coefficients in our models control for all unmeasured and/or omitted person‐level characteristics that may confound the relationship between insurance instability and our outcomes, thus isolating effects that are attributable directly to changes in the independent variable. In the current study, that means that the within‐person effects represent potential causal links between insurance instability and sexual and reproductive health care utilization and contraceptive access. Meanwhile, between‐person effects are typically larger in magnitude because the unmeasured confounding is not removed.

In addition to including time‐invariant characteristics of race/ethnicity and nativity in our hybrid models, we use robust standard errors and adjusted for year, state of residence, age group, LGBTQ identity/orientation, marriage, employment, education, sexual activity in the past 3 months, and desire to avoid pregnancy, each as described previously. Further information on all variable construction is available in our methodological Appendix S1.

This type of model specification does not allow for survey weights and thus we did not incorporate weights into the analysis for our second research aim. In our results, we compare the within and between‐person effects of insurance instability on the odds of each of our binary outcomes. Using random effects for race/ethnicity and nativity, we also highlight the relative odds of experiencing one of these outcomes for racially or ethnically minoritized individuals compared to non‐Hispanic White respondents and among foreign‐born compared to US‐born individuals.

We also tested whether race/ethnicity or nativity modified the relationship between our independent and dependent variables as well as whether the relationship between nativity and our outcomes was modified by race/ethnicity and found no such interactions. Thus, we maintained only the main effects in our final models. Full results tables, including all covariates, are available in Supporting Information.

We conducted all analyses in Stata/MP 18.0.

## 3 Results

### 3.1 Sample Characteristics

Table 1 describes the baseline characteristics of our sample, stratified by race/ethnicity and nativity. Overall, our US‐born sample was younger than our foreign‐born sample. The age gap was widest for Hispanic respondents, among whom 40% of the US‐born respondents were 25 years of age or younger at baseline and thus more likely to still be eligible for coverage under their parents' health insurance, compared to only 16% of their foreign‐born counterparts.

**TABLE 1:** Sample characteristics at first observation, by race/ethnicity and nativity.

		Non‐hispanic white		Hispanic		Other non‐hispanic BIPOC^a^		Total	
		US born	Foreign born	US born	Foreign born	US born	Foreign born	US born	Foreign born
		%;μ	%;μ	%;μ	%;μ	%;μ	%;μ	%;μ	%;μ
State	Arizona	22.7	10.5	58.0	47.9	20.6	12.0	28.4	25.2
	Iowa	28.2	56.5	31.2	44.3	48.5	72.6	31.8	58.1
	New Jersey	29.7	20.8	7.8	5.3	21.2	8.7	24.7	10.3
	Wisconsin	19.4	12.2	3.0	2.5	9.7	6.8	15.1	6.4
Age groups	18–25	27.5	28.2	40.3	15.5	28.8	21.4	29.9	20.8
	26–34	35.8	31.6	33.1	40.3	41.7	28.5	36.2	33.7
	35+	36.7	40.1	26.7	44.2	29.5	50.0	33.9	45.5
Educational attainment	High school or less	13.3	7.7	23.7	27.7	23.9	10.0	16.7	16.2
	Some college	42.9	41.3	51.8	46.7	41.4	38.8	44.2	42.4
	Bachelor's+	43.8	51.0	24.5	25.7	34.6	51.2	39.1	41.5
Employment	Employed	78.7	69.9	73.9	63.9	75.2	72.7	77.3	68.7
	Not employed	21.3	30.1	26.1	36.1	24.8	27.3	22.7	31.3
Marital status	Currently married	45.6	56.9	30.5	48.5	27.4	57.0	40.3	53.7
	Not married	54.4	43.1	69.5	51.5	72.6	43.0	59.7	46.3
LGBTQ + status	Cis/straight	87.0	91.4	78.6	87.7	81.1	90.1	84.7	89.5
	LGBTQ^b^+	13.0	8.6	21.4	12.3	18.9	9.9	15.3	10.5
Had PIV sex in the past 3 month	No	22.5	19.7	24.4	21.4	29.7	34.3	23.9	26.0
	Yes	77.5	80.3	75.6	78.6	70.3	65.7	76.1	74.0
Mean desire to avoid pregnancy score (0 = Low; 4 = High)		2.4	2.5	2.5	2.7	2.6	2.7	2.5	2.6
Received any sexual and reproductive health care in the previous 12 months	No	26.9	35.9	31.7	36.1	32.6	32.0	28.6	34.5
	Yes	73.1	64.1	68.3	63.9	67.4	68.0	71.4	65.5
Received contraceptive care in the previous 12 months	No	48.6	58.9	50.6	56.5	55.6	57.5	50.0	57.5
	Yes	51.4	41.1	49.4	43.5	44.4	42.5	50.0	42.5
Had trouble getting wanted contraception in the previous 12 months	No	89.2	88.9	80.2	77.8	87.7	89.5	87.5	84.9
	Yes	10.8	11.1	19.8	22.2	12.3	10.5	12.5	15.1
Currently using any method of contraception	No	20.9	27.9	26.6	24.9	35.0	32.1	24.0	28.4
	Yes	79.1	72.1	73.4	75.1	65.0	67.9	76.0	71.6
Insurance coverage	Public	12.3	8.2	23.7	15.5	30.8	10.6	17.1	11.9
	Employer‐provided	62.0	55.1	41.5	33.2	40.3	50.9	55.2	45.2
	Self‐paid private	8.9	12.9	6.0	11.1	8.7	7.5	8.4	10.2
	Multiple/other	11.2	15.0	20.1	12.2	16.1	22.7	13.4	16.9
	No insurance	5.6	8.8	8.7	27.9	4.0	8.3	5.9	15.8
Unweighted *N* (first observations only)		3372	82	487	121	371	125	4230	328

^a^ Black indigenous or person of color (i.e., non‐White and non‐Hispanic).

^b^ Lesbian, gay, bisexual, transgender, or queer sexual orientation and/or gender identity.

Most respondents were employed at baseline (64%–79%), with foreign‐born Hispanic respondents having the lowest proportion employed at baseline. Educational attainment varied substantially by race/ethnicity and nativity. Foreign‐born non‐Hispanic White and BIPOC achieved the highest levels of education, with 51% in each group obtaining at least a Bachelor's degree and just 8% of foreign‐born non‐Hispanic White respondents and 10% of non‐Hispanic BIPOC respondents having a high school education or less at baseline. Hispanic respondents had the lowest educational attainment, among whom 24% of US‐born and 28% of foreign‐born respondents had just a high school education or less at baseline.

Marriage was more common for foreign‐born than US‐born respondents. For non‐Hispanic BIPOC respondents in particular, marriage was over twice as common among foreign born (57%) compared to US‐born (27%) respondents. Across all subgroups, most respondents were sexually active (66%–80%) and using some form of contraception (65%–79%) at baseline.

Among all racial/ethnic and nativity subgroups, the most common type of health insurance at baseline was employer‐provided private insurance, covering the majority of our overall US‐born sample (55%) and a large minority of our overall foreign‐born sample (45%). Among US‐born respondents, having public health insurance was considerably more common among Hispanic (24%) and non‐Hispanic BIPOC (31%) respondents compared to their non‐Hispanic White counterparts (12%). Uninsurance at baseline was highest among Hispanic respondents, particularly for those born outside the US, among whom over 1 in 4 reported not having any health insurance coverage at baseline, compared to less than 1 in 10 among their US‐born counterparts and non‐Hispanic foreign‐born counterparts and 1 in 20 among non‐Hispanic US‐born respondents.

### 3.2 Risk of Insurance Instability, by Race/Ethnicity and Nativity

**FIGURE 1:**
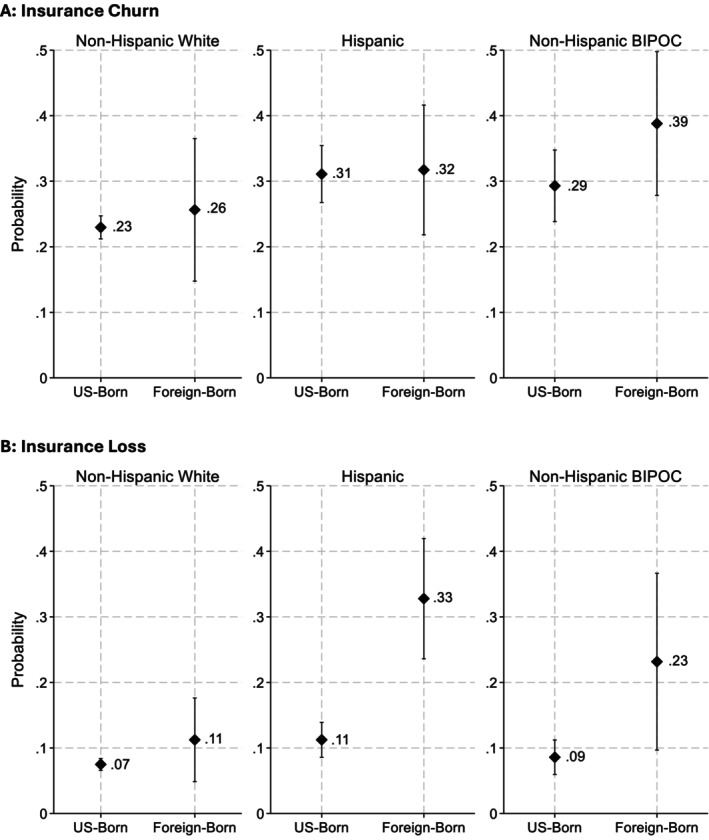
Predicted Probability of Insurance Churn and Loss, by Race/Ethnicity and Nativity. (A): Insurance Churn. (B): Insurance Loss. Generated from our fully‐adjusted population averaged logistic regression models, Figure 1A provides the predicted probability of insurance churn and Figure 1B provides the predicted probability of insurance loss. Each predicted probability graph provided the probability that *Y* = 1 among US‐born respondents vs. Foreign‐born respondents, net of differences in age group, educational attainment, employment, sexual or gender minority status (i.e., non‐straight or cis‐gendered), marital status, state, and year. BIPOC stands for Black, indigenous, or person of color (i.e., non‐White and non‐Hispanic).

Figure 1A provides the predicted probabilities, generated from population‐averaged logistic regressions, of transitioning from one type of insurance to another without experiencing a gap in coverage (i.e., insurance churn) and, despite being a relatively common occurrence, we did not find differential risks across race/ethnicity or nativity. While foreign‐born non‐Hispanic BIPOC respondents did experience more insurance churn during the study period than their US‐born counterparts (39% vs. 29%), the difference was not statistically significant (OR = 1.56; *p* > 0.10). Nativity was also not associated with differential risks of insurance churn within racial groups for White non‐Hispanic nor Hispanic respondents.

However, Figure 1B reveals that Hispanic respondents born outside the United States experienced three times the risk of losing their health insurance coverage compared to their US‐born counterparts (31% vs. 11%; OR = 4.43, *p* < 0.001). Similarly, foreign‐born respondents racialized as non‐Hispanic BIPOC experienced over 2.5 times the risk of losing their health insurance compared to their US‐born counterparts (23% vs. 9%; OR = 3.55, *p* < 0.05). Among White non‐Hispanic respondents, on the other hand, having been born outside the US was not significantly associated with the risk of insurance loss.

### 3.3 Effect of Insurance Instability on Health Service Utilization

Table 2 provides the marginal effect of experiencing insurance churn on the predicted probability of subsequent sexual and reproductive health service utilization and contraceptive access outcomes, and Table 3 highlights the marginal effects of losing health insurance coverage on these outcomes among people who were previously insured.

**TABLE 2:** Marginal effects of insurance churn, nativity, and race/ethnicity on the predicted probability of sexual and reproductive health care and contraceptive access from hybrid within‐between logistic regression models.

	Model 1: received any sexual and reproductive health care		Model 2: received contraceptive care		model 3: difficulty/delay obtaining wanted contraception		Model 4: current use of contraception	
	∆*p*	SE	∆*p*	SE	∆*p*	SE	∆*p*	SE
Insurance churn								
Within‐person effect	−0.005	(0.017)	0.004	(0.016)	0.012	(0.010)	−0.017	(0.012)
Between‐person effect	−0.009	(0.019)	−0.002	(0.019)	0.003	(0.008)	0.007	(0.013)
Random effects								
Foreign‐born (vs. US Born)	−0.020	(0.029)	−0.018	(0.029)	−0.006	(0.013)	0.004	(0.021)
Hispanic (vs. non‐Hispanic)	−0.032	(0.021)	−0.041*	(0.022)	0.015	(0.009)	−0.007	(0.015)
non‐Hispanic BIPOC^a^ (vs. White or Hispanic)	−0.041*	(0.022)	−0.052**	(0.022)	0.020**	(0.009)	−0.018	(0.016)

*Note:* Marginal effects were generated from the coefficients of four hybrid logistic regression models constructed using the multi‐level modeling framework *meglm* and post‐estimation command *margins* in Stata/MP 18. All models control for year, state of residence, age group, baseline sexual or gender minority status (i.e., non‐straight or cis‐gendered), marriage, employment, baseline education, sexual activity in the past 3 months, and desire to avoid pregnancy. Full regression output is available in Supporting Information.

^a^ Black, indigenous, or person of color (i.e., non‐White and non‐Hispanic).

* *p* < 0.10.

** *p* < 0.05.

**TABLE 3:** Marginal effects of insurance loss, nativity, and race/ethnicity on the predicted probability of sexual and reproductive health care and contraceptive access from hybrid within‐between logistic regression models.

	Model 1: Received any sexual and reproductive health care		Model 2: Received contraceptive care		Model 3: Difficulty/delay obtaining wanted contraception		Model 4: Current use of contraception	
	∆*p*	SE	∆*p*	SE	∆*p*	SE	∆*p*	SE
Insurance loss								
Within‐person effect	−0.046**	(0.020)	−0.007	(0.020)	0.053***	(0.010)	−0.003	(0.013)
Between‐person effect	−0.199***	(0.024)	−0.187***	(0.025)	0.071***	(0.010)	−0.045**	(0.017)
Random effects								
Foreign‐born (vs. US Born)	0.007	(0.022)	−0.007	(0.025)	0.000	(0.011)	−0.006	(0.017)
Hispanic (vs. non‐Hispanic)	−0.027	(0.017)	−0.029	(0.019)	0.024**	(0.008)	−0.011	(0.013)
Non‐Hispanic BIPOc^a^ (vs. White or Hispanic)	−0.037**	(0.018)	−0.044**	(0.019)	0.015	(0.009)	−0.019	(0.014)

*Note:* Marginal effects were generated from the coefficients of four hybrid logistic regression models constructed using the multi‐level modeling framework *meglm* and post‐estimation command *margins* in Stata/MP 18. All models control for year, state of residence, age group, baseline sexual or gender minority status (i.e., non‐straight or cis‐gendered), marriage, employment, baseline education, sexual activity in the past 3 months, and desire to avoid pregnancy. Full regression output is available in Supporting Information.

^a^ Black, indigenous, or person of color (i.e., non‐White and non‐Hispanic).

** *p* < 0.05.

*** *p* < 0.001.

#### 3.3.1 Insurance Churn

Our findings in Table 2 suggest that there are no significant differences in sexual and reproductive health service utilization and contraceptive access among people in the wake of insurance churn compared to times when they maintained the same insurance coverage (i.e., within‐person effects) nor between people who did and did not experience insurance churn (i.e., between‐person effects).

#### 3.3.2 Insurance Loss

Table 3 summarizes the marginal effect of losing health insurance coverage on subsequent sexual and reproductive health care utilization and contraceptive access, generated from our hybrid logistic regression models. The between‐person effects from these models suggest that insurance loss is associated with significantly lower sexual and reproductive health and contraceptive care utilization, significantly higher barriers to obtaining contraception, and much lower odds of using contraception among individuals who lose insurance compared to individuals who remain continuously insured. Meanwhile, the within‐person effects, which represent the remaining effect of losing insurance once all time‐invariant individual‐level confounding is removed, indicate that insurance loss reduces the probability of subsequent sexual and reproductive health care utilization by 5 percentage points and increases the odds of experiencing subsequent barriers to obtaining preferred contraception by 5 percentage points, but is no longer associated with subsequent receipt of contraceptive care or use of contraception. Differences between our within and between‐coefficients suggest that there are unmeasured individual‐level factors that both shape the risk of insurance loss and impede sexual and reproductive health care utilization and contraceptive access. However, even after controlling for those unmeasured factors, a person in the wake of insurance loss will have lower odds of utilizing sexual and reproductive health care and a greater chance of experiencing barriers to contraception than they would if they had been continuously covered.

### 3.4 Sexual and Reproductive Health Service Utilization and Contraceptive Access by Race/Ethnicity and Nativity

There were no significant differences across outcomes in Tables 2 and 3 between US‐born and foreign‐born respondents after controlling for covariates.

There was some association between racial/ethnic minoritization and sexual and reproductive health care utilization and contraceptive access that was not explained by differential insurance instability, nativity, and other control variables. Table 3 suggests that non‐Hispanic BIPOC respondents had significantly lower probabilities of receiving any sexual and reproductive health or contraceptive care, and a higher probability of experiencing barriers to obtaining preferred contraception compared to non‐Hispanic White or Hispanic respondents, net of insurance instability, nativity, and other covariates.

Similarly, Table 2 suggests that Hispanic respondents had a lower probability of receiving contraceptive care, net of insurance churn, nativity, and other covariates. Table 3 suggests that Hispanic respondents also had a higher probability of experiencing a barrier to obtaining contraception than non‐Hispanic white respondents, net of insurance loss, nativity, and other covariates.

## 4 Discussion

### 4.1 Summary of Findings

Consistent with existing research, this study finds that insurance churn is relatively common and widespread across all racial/ethnic and nativity subgroups, with predicted probabilities of experiencing a change in insurance ranging from 23% of US‐born non‐Hispanic White to 39% of foreign‐born non‐Hispanic BIPOC respondents.

While limited evidence suggests that insurance churn may increase disruptions in care, even without gaps in coverage [12, 19], we did not detect any significant differences in sexual and reproductive health care utilization or contraceptive access outcomes for people who remained continuously insured but changed insurance type. Notably, our study uses a different target population, methodological approach, and different outcomes of interest than the two known quantitative studies investigating the consequences of insurance churn separately from insurance loss [12, 28]. These studies both show an increase in emergency department visits and more switching of providers following insurance churn, neither of which is an outcome we measure in our study. In fact, our study measures only whether care was received and not where or from whom, so while we might expect to see an increase in reports of barriers to care following an insurance churn, if people are still seeking care but changing where and how they obtain it under a new plan, we would not see a change to overall levels of utilization.

In contrast to insurance churn, we found that insurance loss was markedly more common for foreign‐born compared to US‐born respondents. Foreign‐born Hispanic respondents were particularly vulnerable to insurance loss, experiencing a probability of losing insurance three times that of our US‐born Hispanic respondents. Similarly, among non‐Hispanic BIPOC respondents, we estimated that those born outside the US experienced insurance loss over 2.5 times more often than their US‐born counterparts. Meanwhile, consistent with existing evidence on the negative consequences of insurance loss for access to care [12, 13, 18, 20], the findings from our hybrid models showed that losing insurance was a strong hindrance to sexual and reproductive healthcare utilization and contraceptive access, resulting in lower sexual and reproductive health care utilization and more frequent barriers to obtaining contraception, but no decline in utilization of contraceptive care or use of contraception. Taken together, these findings suggest that, in the wake of insurance loss, people may make tough decisions about what care to prioritize and what to sacrifice, perhaps prioritizing pregnancy prevention goals by continuing to use contraception even if they have difficulty accessing desired methods and deprioritizing other less immediately salient health goals by foregoing broader preventative sexual and reproductive health care. That we do not see a suppression of overall contraceptive use speaks to the notion that contraception is a priority for women wishing to avoid pregnancy, but the heightened barriers experienced in obtaining wanted methods is an important indicator of how important insurance coverage is for facilitating access to preferred methods. Qualitative findings from the Reproductive Health Impact Study add corroborating evidence both that changes in insurance lead people to delay or forego care [11] and that contraceptive users who lose insurance coverage may substitute for less preferred contraceptive methods [38]. Furthermore, the disproportionate burden of insurance loss among immigrant people of color in our study suggests that these tough decisions surrounding sexual and reproductive health care utilization and barriers to contraceptive access are even more common for a population that already experiences relatively high barriers to obtaining this care due to the overlapping systems of oppression and structural disadvantage they face relative to US‐born White people.

### 4.2 Limitations

This analysis uses nativity to compare the experiences of people born outside the United States to those who are native born. Nativity, however, does not denote current immigration status, which may be a better proxy for understanding the administrative barriers and social stressors experienced by people with various immigration statuses. We also have no information related to acculturation (e.g., duration in the US, languages spoken at home, etc.) and thus do not control for these factors in our models.

Additionally, our sample of foreign‐born respondents was quite small, with 328 foreign‐born respondents at baseline, which restricted our ability to disaggregate by both race/ethnicity and state of residence in addition to nativity. As state and federal policies likely shape access to coverage and care, particularly for immigrants of color, this disaggregation would be a useful inclusion in future studies with more observations. The small foreign‐born sample may have also prevented us from being able to detect statistically significant differences in the risk of insurance churn by nativity and thus to more soundly falsify alternative hypotheses regarding the relationship between insurance churn and sexual and reproductive healthcare utilization and contraceptive access.

Furthermore, we note that our US‐born sample is considerably younger than our foreign‐born sample and thus may be more likely to have obtained insurance coverage from a parent's employer‐provided health plan. As US‐born respondents are already more likely to be insured under an employer‐provided insurance plan, this might represent a fundamental difference in the insurance patterns of these two samples that could be better captured in future studies by explicitly capturing insurance obtained through family members' employment.

We also note two limitations in how we operationalize insurance instability. First, we have a limited number of follow‐up observations. Future studies with more intermediate observations may better capture churn and its consequences. Secondly, our research question and methodological approach do not capture the unique vulnerabilities experienced by people who are continuously uninsured, for whom barriers to sexual and reproductive health care utilization and contraceptive access are likely to be particularly high.

Lastly, while the within‐person coefficients in our hybrid models remove unmeasured level‐two (time‐invariant) confounding of the relationship between insurance instability and our outcomes, allowing for causal interpretations of the relationship between insurance instability and our outcomes, those causal interpretations assume no other unmeasured level‐one changes confound that relationship across time.

### 4.3 Implications

This study reconfirms the important role continuous and reliable health insurance coverage plays in protecting access to sexual and reproductive health care and promoting sexual and reproductive health and well‐being. However, while insurance churn is a common occurrence that previous research has linked to disruptions in care, our study suggests that churn without insurance loss may not pose the same risk to sexual and reproductive health care utilization and contraceptive access that is experienced by people who lose coverage altogether. Furthermore, while the risk of insurance churn is widespread, people born outside the US, particularly those who are racially or ethnically minoritized, experience a higher risk of insurance loss. Future research should investigate the extent to which disparities in sexual and reproductive health outcomes may be directly attributable to disparate risks of insurance instability for certain marginalized groups and what other factors may be at play that are correlated with but distinct from insurance loss.

While expanding access to continuous and reliable coverage should be a sexual and reproductive health equity priority, the publicly funded sexual and reproductive health care system is an important safety net for the underinsured and uninsured. Title X sites are a particularly important access point for immigrants seeking access to contraception and related care [39]. Higher levels of uninsurance among immigrants and their families stem from both real immigration‐related eligibility barriers and/or under‐utilization of available public insurance benefits because of confusion or fear [22, 23, 24]. Furthermore, non‐citizen immigrants, particularly those who are racially minoritized, are more frequently employed in lower‐paying jobs without benefits [23]. Thus, for immigrants of color, structural nativism and racism jointly create barriers to accessing health promoting resources like insurance and obstruct common pathways to coverage, such as higher‐paying, more secure jobs. These overlapping systems of structural disadvantage threaten sexual and reproductive health equity and highlight the need for government policy, healthcare systems, and other structures that aim to promote sexual and reproductive health and well‐being and sexual and reproductive health equity to reckon with the sociopolitical forces and historical context that produce unequal outcomes along lines of race/ethnicity and nativity in the United States. In light of ongoing threats to health and social programs and the hostile immigration enforcement policies and procedures enacted under the Trump‐Vance administration [40, 41], advocating for concrete policies that protect sexual and reproductive health equity is crucially important.

## Conflicts of Interest

The authors declare no conflicts of interest.

## Supporting information


**Table S1:** hesr70049‐sup‐0001‐Tables.docx.

**Appendix S1:** hesr70049‐sup‐0002‐AppendixS1.docx.

## Data Availability

The data that support the findings of this study are available on request from the corresponding author. The data are not publicly available due to privacy or ethical restrictions.
