# Supplementary material for: How Health Insurance Instability Differentially Impedes Access to Sexual and Reproductive Healthcare, by Race/Ethnicity and Nativity
Source: Health Serv Res. 2025 Oct 7;61(1):e70049. doi: 10.1111/1475-6773.70049 (PMC12857480; doi:10.1111/1475-6773.70049)
Supplement: Supplementary file 1 — Table S1: hesr70049‐sup‐0001‐Tables.docx. [file HESR-61-e70049-s002.docx]

| **Appendix Table 1: Relative odds of changing insurance among those who are continuously insured, by nativity and race/ethnicity** | | | | | | | | | | |
| --- | --- | --- | --- | --- | --- | --- | --- | --- | --- | --- |
|  |  | **White non-Hispanic** | | | **Hispanic/Latinx** | | | **Other BIPOC** | | |
| **Category** |  | aOR |  | 95% CI | aOR |  | 95% CI | aOR |  | 95% CI |
| **Nativity** | Born in the US (ref.) | 1.00 |  | -- | 1.00 |  | -- | 1.00 |  | -- |
|  | Born outside the US | 1.16 |  | (0.63-2.14) | 1.03 |  | (0.60-1.77) | 1.56 |  | (0.87-2.80) |
| **Age Groups** | 18-25 (ref) | 1.00 |  |  | 1.00 |  | -- | 1.00 |  | -- |
|  | 26-34 | 0.67 | ** | (0.47-0.95) | 0.34 | *** | (0.19-0.62) | 0.73 |  | (0.38-1.41) |
|  | 35+ | 0.45 | *** | (0.32-0.63) | 0.28 | *** | (0.16-0.50) | 0.48 | * | (0.23-1.00) |
| **Education** | High School or Less (ref) | 1.00 |  | -- | 1.00 |  | -- | 1.00 |  | -- |
|  | Some College or Associates | 1.20 |  | (0.83-1.72) | 1.19 |  | (0.67-2.11) | 1.04 |  | (0.52-2.10) |
|  | Bachelor's or Higher | 0.68 | ** | (0.47-0.97) | 0.72 |  | (0.39-1.33) | 0.80 |  | (0.39-1.62) |
| **Employed (vs. Not)** | | 0.98 |  | (0.69-1.39) | 1.38 |  | (0.74-2.57) | 0.83 |  | (0.44-1.57) |
| **LGBTQ+ (vs. Cis/Straight)** | | 0.86 |  | (0.60-1.23) | 0.71 |  | (0.43-1.16) | 1.29 |  | (0.69-2.41) |
| **Married (vs. Not)** | | 0.96 |  | (0.76-1.21) | 1.37 |  | (0.82-2.28) | 0.93 |  | (0.54-1.60) |
| **State** | Arizona (ref) | 1.00 |  | -- | 1.00 |  | -- | 1.00 |  | -- |
|  | Iowa | 0.93 |  | (0.66-1.31) | 0.93 |  | (0.58-1.49) | 0.67 |  | (0.38-1.19) |
|  | Wisconsin | 0.79 |  | (0.57-1.08) | 0.63 |  | (0.28-1.44) | 0.80 |  | (0.39-1.64) |
|  | New Jersey | 0.95 |  | (0.70-1.29) | 0.94 |  | (0.46-1.93) | 0.95 |  | (0.48-1.87) |
| **Year** | 2020 (ref) | 1.00 |  | -- | 1.00 |  | -- | 1.00 |  | -- |
|  | 2021 | 0.99 |  | (0.79-1.26) | 1.06 |  | (0.43-2.63) | 0.83 |  | (0.41-1.68) |
|  | 2022 | 0.73 | ** | (0.57-0.93) | 0.79 |  | (0.31-1.98) | 0.99 |  | (0.47-2.09) |
| **Intercept** |  | 0.68 |  | (0.41-1.14) | 0.86 |  | (0.27-2.80) | 1.09 |  | (0.37-3.20) |
| Number of Observations | | 6207 | | | 862 | | | 788 | | |
| Number of Respondents | | 3185 | | | 503 | | | 440 | | |
| * p<0.10; ** p<0.05; *** p<0.001 | |  |  |  |  |  |  |  |  |  |

Odds ratios are generated from population averaged logistic regression models of insurance churn using Stata’s xtlogit command.

| **Appendix Table 2: Relative odds of losing insurance vs. remaining insured, by nativity and race/ethnicity** | | | | | | | | | | |
| --- | --- | --- | --- | --- | --- | --- | --- | --- | --- | --- |
|  |  | **White non-Hispanic** | | | **Hispanic/Latinx** | | | **Other BIPOC** | | |
| **Category** |  | aOR |  | 95% CI | aOR |  | 95% CI | aOR |  | 95% CI |
| **Nativity** | Born in the US (ref.) | 1.00 |  | -- | 1.00 |  | -- | 1.00 |  | -- |
|  | Born outside the US | 1.59 |  | (0.80-3.13) | 4.43 | *** | (2.45-8.02) | 3.55 | ** | (1.32-9.57) |
| **Age Groups** | 18-25 (ref) | 1.00 |  | -- | 1.00 |  | -- | 1.00 |  | -- |
|  | 26-34 | 1.73 | ** | (1.16-2.58) | 1.28 |  | (0.66-2.46) | 2.56 | ** | (1.06-6.19) |
|  | 35+ | 1.13 |  | (0.74-1.74) | 0.43 | ** | (0.20-0.91) | 1.45 |  | (0.39-5.39) |
| **Education** | High School or Less (ref) | 1.00 |  | -- | 1.00 |  | -- | 1.00 |  | -- |
|  | Some College or Associates | 1.03 |  | (0.68-1.56) | 0.79 |  | (0.43-1.44) | 0.63 |  | (0.24-1.62) |
|  | Bachelor's or Higher | 0.68 | * | (0.44-1.05) | 0.65 |  | (0.34-1.25) | 0.38 | * | (0.14-1.02) |
| **Employed (vs. Not)** | | 0.99 |  | (0.70-1.41) | 0.78 |  | (0.50-1.22) | 0.69 |  | (0.33-1.41) |
| **LGBTQ+ (vs. Cis/Straight)** | | 1.37 | * | (0.97-1.95) | 2.29 | ** | (1.31-4.01) | 0.92 |  | (0.40-2.14) |
| **Married (vs. Not)** | | 0.62 | ** | (0.46-0.85) | 0.53 | ** | (0.30-0.92) | 0.30 | ** | (0.11-0.79) |
| **State** | Arizona (ref) | 1.00 |  | -- | 1.00 |  | -- | 1.00 |  | -- |
|  | Iowa | 0.65 | ** | (0.45-0.94) | 0.69 |  | (0.38-1.27) | 1.36 |  | (0.60-3.09) |
|  | Wisconsin | 0.57 | ** | (0.39-0.82) | 1.87 |  | (0.80-4.33) | 1.21 |  | (0.46-3.18) |
|  | New Jersey | 0.35 | *** | (0.25-0.49) | 0.62 |  | (0.30-1.31) | 1.24 |  | (0.47-3.29) |
| **Year** | 2018 (ref) | 1.00 |  | -- | 1.00 |  | -- | 1.00 |  | -- |
|  | 2019 | 2.02 | ** | (1.09-3.73) | 0.38 |  | (0.10-1.40) | 1.44 |  | (0.53-3.94) |
|  | 2020 | 1.56 |  | (0.88-2.75) | 0.33 | * | (0.10-1.07) | 0.94 |  | (0.44-2.00) |
|  | 2021 | 0.99 |  | (0.55-1.79) | 0.24 | ** | (0.07-0.80) | 0.89 |  | (0.42-1.90) |
|  | 2022 | 0.80 |  | (0.44-1.45) | 0.20 | ** | (0.06-0.68) | 0.66 |  | (0.28-1.56) |
| **Intercept** |  | 0.11 | *** | (0.05-0.24) | 0.85 |  | (0.24-3.06) | 0.15 | ** | (0.05-0.50) |
| Number of Observations | | 9359 | | | 1475 | | | 1253 | | |
| Number of Respondents | | 3450 | | | 603 | | | 496 | | |
| * p<0.10; ** p<0.05; *** p<0.001 | |  |  |  |  |  |  |  |  |  |

Odds ratios are generated from population averaged logistic regression models of insurance loss using Stata’s xtlogit command.

| **Appendix Table 3:** Within-between logistic regressions of insurance churn on four sexual and reproductive health care utilization and contraceptive access outcomes | | | | | | | | | | | | |
| --- | --- | --- | --- | --- | --- | --- | --- | --- | --- | --- | --- | --- |
|  | Received any sexual and reproductive health care | | | Received contraceptive care | | | Experienced barriers to obtaining wanted contraception | | | Current use of contraception | | |
|  | aOR |  | (95% CI) | aOR |  | (95% CI) | aOR |  | (95% CI) | aOR |  | (95% CI) |
| Random effects |  |  |  |  |  |  |  |  |  |  |  |  |
| Foreign-born (vs. US Born) | 0.84 |  | (0.51 - 1.38) | 0.84 |  | (0.49 - 1.45) | 0.85 |  | (0.46 - 1.59) | 1.08 |  | (0.52 - 2.22) |
| Hispanic (vs. Non-Hispanic) | 0.75 |  | (0.52 - 1.09) | 0.68 | * | (0.45 - 1.01) | 1.46 |  | (0.93 - 2.31) | 0.88 |  | (0.53 - 1.46) |
| Non-Hispanic BIPOC (vs. Hispanic and White) | 0.69 | * | (0.47 - 1.03) | 0.61 | ** | (0.41 - 0.92) | 1.65 | ** | (1.04 - 2.62) | 0.73 |  | (0.42 - 1.26) |
| Education: Less than high school diploma | 0.78 |  | (0.52 - 1.17) | 0.80 |  | (0.51 - 1.25) | 0.86 |  | (0.52 - 1.43) | 0.89 |  | (0.51 - 1.58) |
| Education: Bachelor's degree or higher | 1.52 | ** | (1.17 - 1.96) | 1.56 | ** | (1.19 - 2.04) | 0.96 |  | (0.69 - 1.34) | 1.57 | ** | (1.09 - 2.24) |
| LGBTQ Identify/Orientation | 0.76 |  | (0.54 - 1.08) | 0.46 | *** | (0.31 - 0.67) | 1.81 | ** | (1.23 - 2.67) | 0.35 | *** | (0.22 - 0.55) |
| Arizona resident | 0.66 | ** | (0.45 - 0.96) | 0.55 | ** | (0.37 - 0.82) | 1.48 |  | (0.91 - 2.41) | 0.86 |  | (0.51 - 1.46) |
| New Jersey resident | 1.12 |  | (0.77 - 1.61) | 0.74 |  | (0.50 - 1.10) | 1.34 |  | (0.84 - 2.15) | 0.69 |  | (0.42 - 1.13) |
| Iowa resident | 0.79 |  | (0.55 - 1.12) | 0.61 | ** | (0.42 - 0.89) | 0.88 |  | (0.54 - 1.44) | 1.34 |  | (0.81 - 2.22) |
| Within-person effects |  |  |  |  |  |  |  |  |  |  |  |  |
| Insurance churn | 0.95 |  | (0.71 - 1.27) | 1.04 |  | (0.77 - 1.40) | 1.36 |  | (0.84 - 2.20) | 0.75 |  | (0.49 - 1.14) |
| Age: 18-25 years | 0.72 |  | (0.34 - 1.53) | 0.47 | ** | (0.23 - 0.94) | 0.95 |  | (0.33 - 2.78) | 0.93 |  | (0.29 - 2.99) |
| Age: 35-44 years | 1.29 |  | (0.67 - 2.5) | 1.47 |  | (0.76 - 2.83) | 2.16 |  | (0.65 - 7.19) | 1.63 |  | (0.65 - 4.09) |
| Employed | 0.95 |  | (0.56 - 1.61) | 1.38 |  | (0.81 - 2.35) | 1.07 |  | (0.49 - 2.34) | 0.89 |  | (0.39 – 2.00) |
| Married | 1.38 |  | (0.75 - 2.56) | 1.06 |  | (0.56 - 2.01) | 0.47 |  | (0.15 - 1.45) | 0.62 |  | (0.26 - 1.49) |
| PIV sex in preceding 3 months | 1.19 |  | (0.76 - 1.86) | 1.31 |  | (0.81 - 2.11) | 1.02 |  | (0.49 - 2.10) | 21.65 | *** | (10.28 - 45.59) |
| DAP score | 0.98 |  | (0.79 - 1.23) | 0.72 | ** | (0.57 - 0.91) | 0.77 |  | (0.54 - 1.11) | 0.42 | *** | (0.31 - 0.58) |
| Year | 0.83 | ** | (0.74 - 0.94) | 0.75 | *** | (0.66 - 0.85) | 0.73 | ** | (0.59 - 0.91) | 1.05 |  | (0.87 - 1.26) |
| Between-person effects |  |  |  |  |  |  |  |  |  |  |  |  |
| Insurance churn | 0.92 |  | (0.67 - 1.28) | 0.98 |  | (0.69 - 1.38) | 1.09 |  | (0.73 - 1.62) | 1.13 |  | (0.72 - 1.79) |
| Age: 18-25 years | 1.13 |  | (0.75 - 1.72) | 1.23 |  | (0.80 - 1.90) | 1.52 | * | (0.98 - 2.35) | 0.71 |  | (0.41 - 1.24) |
| Age: 35-44 years | 0.15 | *** | (0.11 - 0.20) | 0.18 | *** | (0.13 - 0.25) | 0.31 | *** | (0.21 - 0.45) | 1.02 |  | (0.71 - 1.45) |
| Employed | 1.78 | ** | (1.24 - 2.54) | 2.10 | *** | (1.44 - 3.07) | 0.46 | *** | (0.29 - 0.72) | 1.40 |  | (0.88 - 2.24) |
| Married | 0.40 | *** | (0.30 - 0.52) | 0.27 | *** | (0.20 - 0.37) | 0.49 | *** | (0.33 - 0.71) | 0.76 |  | (0.52 - 1.12) |
| PIV sex in preceding 3 months | 4.46 | *** | (3.17 - 6.28) | 4.35 | *** | (3.00 - 6.29) | 2.51 | *** | (1.64 - 3.84) | 689.64 | *** | (320.68 - 1483.08) |
| DAP score | 1.17 | ** | (1.05 - 1.31) | 0.69 | *** | (0.61 - 0.78) | 0.64 | *** | (0.55 - 0.75) | 0.17 | *** | (0.14 - 0.22) |
| Year | 1.49 | ** | (1.01 - 2.19) | 1.65 | ** | (1.07 - 2.55) | 0.83 |  | (0.53 - 1.29) | 2.32 | ** | (1.36 - 3.93) |
| Number of Observations |  |  | 7,857 |  |  | 7,857 |  |  | 7,857 |  |  | 7,857 |
| Number of Respondents |  |  | 4,128 |  |  | 4,128 |  |  | 4,128 |  |  | 4,128 |
| Chi-2 Test Statistic |  |  | 325.1 |  |  | 302.2 |  |  | 189.2 |  |  | 333.1 |

*p<0.10; **p<0.05; ***p<0.001

| **Appendix Table 4:** Within-between logistic regressions of insurance loss on four sexual and reproductive health care utilization and contraceptive access outcomes | | | | | | | | | | | | |
| --- | --- | --- | --- | --- | --- | --- | --- | --- | --- | --- | --- | --- |
|  | Received any sexual and reproductive health care | | | Received contraceptive care | | | Experienced barriers to obtaining wanted contraception | | | Current use of contraception | | |
|  | aOR |  | (95% CI) | aOR |  | (95% CI) | aOR |  | (95% CI) | aOR |  | (95% CI) |
| Random effects |  |  |  |  |  |  |  |  |  |  |  |  |
| Foreign-born (vs. US Born) | 1.06 |  | (0.76 - 1.46) | 0.95 |  | (0.64 - 1.41) | 1.00 |  | (0.65 - 1.52) | 0.92 |  | (0.57 - 1.48) |
| Hispanic (vs. Non-Hispanic) | 0.82 |  | (0.64 - 1.05) | 0.79 |  | (0.59 - 1.07) | 1.60 | ** | (1.18 - 2.16) | 0.86 |  | (0.61 - 1.22) |
| Non-Hispanic BIPOC (vs. Hispanic and White) | 0.76 | ** | (0.58 - 0.99) | 0.70 | ** | (0.51 - 0.95) | 1.34 |  | (0.93 - 1.91) | 0.76 |  | (0.52 - 1.12) |
| Education: Less than high school diploma | 0.81 |  | (0.61 - 1.08) | 0.74 | * | (0.53 - 1.02) | 1.02 |  | (0.72 - 1.44) | 0.85 |  | (0.57 - 1.27) |
| Education: Bachelor's degree or higher | 1.53 | *** | (1.28 - 1.82) | 1.50 | *** | (1.22 - 1.85) | 0.89 |  | (0.7 - 1.13) | 1.51 | ** | (1.16 - 1.95) |
| LGBTQ Identify/Orientation | 0.79 | * | (0.62 - 1) | 0.58 | *** | (0.44 - 0.77) | 1.52 | ** | (1.14 - 2.02) | 0.38 | *** | (0.28 - 0.52) |
| Arizona resident | 0.97 |  | (0.76 - 1.23) | 0.64 | ** | (0.48 - 0.85) | 1.54 | ** | (1.1 - 2.15) | 1.03 |  | (0.73 - 1.46) |
| New Jersey resident | 1.54 | *** | (1.21 - 1.97) | 0.75 | * | (0.56 - 1) | 1.50 | ** | (1.08 - 2.07) | 0.74 | * | (0.53 - 1.04) |
| Iowa resident | 1.14 |  | (0.91 - 1.43) | 0.65 | ** | (0.5 - 0.84) | 1.21 |  | (0.88 - 1.68) | 1.25 |  | (0.9 - 1.74) |
| Within-person effects |  |  |  |  |  |  |  |  |  |  |  |  |
| Insurance loss | 0.71 | ** | (0.53 - 0.95) | 0.94 |  | (0.68 - 1.3) | 2.78 | *** | (1.93 - 4.02) | 0.96 |  | (0.67 - 1.39) |
| Age: 18-25 years | 0.41 | *** | (0.27 - 0.64) | 0.70 |  | (0.45 - 1.07) | 0.56 | ** | (0.32 - 0.96) | 0.87 |  | (0.48 - 1.6) |
| Age: 35-44 years | 0.96 |  | (0.67 - 1.37) | 0.88 |  | (0.6 - 1.29) | 1.27 |  | (0.65 - 2.46) | 1.07 |  | (0.65 - 1.76) |
| Employed | 1.15 |  | (0.89 - 1.49) | 1.22 |  | (0.92 - 1.6) | 1.09 |  | (0.75 - 1.57) | 1.63 | ** | (1.16 - 2.3) |
| Married | 1.21 |  | (0.84 - 1.74) | 0.63 | ** | (0.43 - 0.93) | 0.49 | ** | (0.25 - 0.92) | 0.61 | ** | (0.38 - 0.99) |
| PIV sex in preceding 3 months | 0.99 |  | (0.77 - 1.28) | 1.44 | ** | (1.1 - 1.87) | 1.56 | ** | (1.06 - 2.31) | 18.92 | *** | (12.82 - 27.93) |
| DAP score | 1.03 |  | (0.9 - 1.17) | 0.72 | *** | (0.62 - 0.82) | 0.81 | ** | (0.67 - 0.98) | 0.41 | *** | (0.35 - 0.49) |
| Year | 0.62 | *** | (0.59 - 0.66) | 0.99 |  | (0.94 - 1.05) | 0.71 | *** | (0.65 - 0.78) | 1.01 |  | (0.94 - 1.09) |
| Between-person effects |  |  |  |  |  |  |  |  |  |  |  |  |
| Insurance loss | 0.23 | *** | (0.16 - 0.33) | 0.22 | *** | (0.14 - 0.33) | 3.94 | *** | (2.7 - 5.74) | 0.53 | ** | (0.34 - 0.85) |
| Age: 18-25 years | 1.12 |  | (0.83 - 1.52) | 1.48 | ** | (1.07 - 2.05) | 1.26 |  | (0.91 - 1.74) | 0.78 |  | (0.53 - 1.15) |
| Age: 35-44 years | 0.28 | *** | (0.23 - 0.34) | 0.24 | *** | (0.19 - 0.3) | 0.36 | *** | (0.28 - 0.48) | 0.88 |  | (0.67 - 1.15) |
| Employed | 1.55 | *** | (1.22 - 1.97) | 1.64 | *** | (1.23 - 2.18) | 0.54 | *** | (0.38 - 0.75) | 1.11 |  | (0.79 - 1.57) |
| Married | 0.55 | *** | (0.45 - 0.67) | 0.36 | *** | (0.29 - 0.46) | 0.50 | *** | (0.38 - 0.66) | 0.95 |  | (0.72 - 1.26) |
| PIV sex in preceding 3 months | 3.80 | *** | (2.97 - 4.86) | 4.39 | *** | (3.25 - 5.93) | 2.56 | *** | (1.85 - 3.53) | 223.03 | *** | (144.8 - 343.54) |
| DAP score | 1.10 | ** | (1.01 - 1.19) | 0.72 | *** | (0.65 - 0.79) | 0.65 | *** | (0.58 - 0.73) | 0.23 | *** | (0.2 - 0.27) |
| Year | 0.72 | *** | (0.61 - 0.86) | 1.18 |  | (0.96 - 1.45) | 0.77 | ** | (0.62 - 0.97) | 2.06 | *** | (1.61 - 2.64) |
| Number of Observations |  |  | 12,208 |  |  | 12,208 |  |  | 12,208 |  |  | 12,208 |
| Number of Respondents |  |  | 4,558 |  |  | 4,558 |  |  | 4,558 |  |  | 4,558 |
| Chi-2 Test Statistic |  |  | 632.32 |  |  | 513.60 |  |  | 443.98 |  |  | 798.28 |

*p<0.10; **p<0.05; ***p<0.001
