# Supplementary material for: How Health Insurance Instability Differentially Impedes Access to Sexual and Reproductive Healthcare, by Race/Ethnicity and Nativity
Source: Health Serv Res. 2025 Oct 7;61(1):e70049. doi: 10.1111/1475-6773.70049 (PMC12857480; doi:10.1111/1475-6773.70049)
Supplement: Supplementary file 2 — Appendix S1: hesr70049‐sup‐0002‐AppendixS1.docx. [file HESR-61-e70049-s001.docx]

Methodological Appendix

Table of Contents

[Analytic Sample Selection 2](#_Toc209692429)

[Variable Construction 3](#_Toc209692430)

[Dependent Variables: Sexual and Reproductive Health Service Utilization and Contraceptive Access 3](#_Toc209692431)

[Independent Variables: Insurance instability 5](#_Toc209692432)

[Other covariates 7](#_Toc209692433)

[State 7](#_Toc209692434)

[Respondent age 7](#_Toc209692435)

[Educational attainment 7](#_Toc209692436)

[Employment 8](#_Toc209692437)

[LGBTQ+ identity/orientation 8](#_Toc209692438)

[Marital status 9](#_Toc209692439)

[Race/ethnicity: 10](#_Toc209692440)

[Nativity 10](#_Toc209692441)

[Desire to Avoid Pregnancy (DAP): 10](#_Toc209692442)

[Other methodological notes 12](#_Toc209692443)

| Analytic Sample Selection | | |
| --- | --- | --- |
| **Inclusions** |  | **Exclusions** |
| **Respondents in Arizona, Iowa, New Jersey and Wisconsin**  (20,053 observations among 8,547 respondents) |  | **Fewer than two consecutive observations with insurance status n = 3, 369 observations/respondents removed**   - Observed only at baseline (lost to follow up) (n= 2,932 resp.) - Missing insurance at baseline (n = 155 resp.) - No two *consecutive* observations (n = 282 resp.) |
|  |  |  |
|  |  |  |
| **Respondents with two consecutive observations of insurance status/type**  (16,097 observations among 5,178 respondents) |  | **Ineligible based on current pregnancy, infertility, or use of sterilization as only method of contraception (17% observations)**  **n = 2,787 observations removed**   - Currently pregnant (n=569 obs.) - Infertile (n = 821 obs.) - Sterilization only users (n = 1,534 obs.) |
|  |  |  |
|  |  |  |
| **Not pregnant. Not infertile. Not sterilized-only users**  (n = 13,310 observations among 4,686 respondents) |  | **Missing responses on key analytic variables (8% observations)**  **n = 1,102 observations removed**   - Sexually active in the last 3 months (missing n=509 obs.) - Marital status (missing n=91 obs.) - Employment (missing n=158 obs.) - Educational attainment (missing n=12 obs.) - Desire to avoid pregnancy [DAP] score (missing n=153 obs.) - SRH services received (missing n=51 obs.) - Contraceptive services received (missing 74 obs.) - Delays or trouble getting birth control (missing n=66 obs.) - Contraceptive use (missing n=112 obs.) - Insurance loss (missing n=132 obs.) |
|  |  |  |
|  |  |  |
|  |  |  |
|  |  |  |
| **Final sample for insurance loss analysis** (n=12,208 observations among 4,558 respondents)  **Final sample for insurance churn analysis** (n=7,857 observations among 4,128 respondents)  Additional exclusions for insurance churn analysis: n = 4351 observations & 430 respondents   - 3,877 baseline observations in the sample for insurance loss analysis, where gaps in insurance coverage over the preceding year were gathered retrospectively but for whom previous insurance type is unknown. - 474 observations for whom insurance churn is missing because the respondent (1) was uninsured at the preceding observation, (2) was missing insurance information at the current or preceding observation, or (3) was not interviewed in the preceding round, leaving a gap longer than 12 months between observations. | | |

# Variable Construction

Note: the question numbers below do not correspond to the numbering of questions in the original questionnaire. The ordering of questions in this appendix also does not correspond to the ordering of questions in the original questionnaire. We provide only the specific questions and answer choices relevant to this analysis in this appendix.

## Dependent Variables: Sexual and Reproductive Health Service Utilization and Contraceptive Access

1. Received any sexual and reproductive health care (RECEIVED SRH CARE)
2. Received contraceptive care (RECEIVED CP CARE)
3. Experienced a barrier to obtaining wanted contraception (BARRIER TO CONTRACEPTION)
4. Currently using any method of contraception (CURRENTLY USING)

#### Relevant survey questions

DV-Q1: In the past 12 months, have you gone to a doctor or other healthcare provider for:

(check all that apply):

1. A method of birth control or a prescription for a birth control method
2. A check-up or medical test related to using a birth control method
3. Counseling or information about birth control
4. A discussion about whether you want to become pregnant in the next year
5. A pregnancy test
6. A general gynecological check-up (e.g., an annual women’s visit)

DV-Q2: In the past 12 months, have you delayed or had trouble getting the birth control method you wanted for any reason?

- - Yes
  - No
  - Prefer not to answer

DV-Q3a In the past 3 months, have you or a partner with whom you have had penile-vaginal sex or sex that could lead to pregnancy used any method or methods of birth control?

- - Yes
  - No
  - Prefer not to answer

*Note: This question wording was used at all time points in Arizona, New Jersey and Wisconsin; it was used in Iowa at follow-up 2 and follow-up 3*

DV-Q3b: Are you currently using any method or methods of birth control?

- - 1. Yes
  - 2. No
  - 99. Prefer not to answer

*Note: This question wording was used in Iowa at baseline and follow-up 1*

DV-Q4: You just indicated that you have NOT used birth control of any kind in the PAST 3 MONTHS. This means you have NOT used anything like pulling out, the calendar method, condoms, a Depo shot, emergency contraception like Plan B®, vasectomy, tubal ligation, or any other method of preventing pregnancy. Is that correct?

- - Correct, I have NOT used birth control
  - Incorrect, I HAVE used birth control
- 77. Don’t know
- 99. Prefer not to answer

*Note: This question was asked in Arizona, New Jersey and Wisconsin in follow-up 1 and follow-up 2; it was asked in Iowa in follow-up 2 and follow-up 3. This question is skipped if respondent answered YES to curruse_yn.*

DV-Q5: What are your reasons for not using any method of birth control?

*Please check all that apply.*

- We just use “pulling out”
- Other, please specify: [a method is specified]

DV-Q6: Are you still using the [IUD type] IUD that you reported using the last time you completed this survey?

- - Yes
  - No
  - Prefer not to answer

#### Variable construction:

- Respondent is considered to have **RECEIVED SRH CARE** if they report receiving any one of the six items listed in DV-Q1 above (1a-1f).
- Respondent is considered to have **RECEIVED CONTRACEPTIVE CARE** if they report receiving a) a method of birth control or a prescription for a birth control method, b) a check-up or medical test related to using a birth control method, or c) counseling or information about birth control in DV-Q1 above.
- Respondent is considered to have experienced a **BARRIER TO CONTRACEPTION** if they respond “Yes” to DV-Q2 above.
- Respondent is considered to be **CURRENTLY USING** contraception if “Yes” is selected for questions 3a or 3b above, or if any of the following are true:
  1. Respondent answered “Incorrect, I HAVE used birth control” to DV-Q4 above.
  2. Respondent selected “We just use ‘pulling out’” or indicated another method being used in response to DV-Q5 above.
  3. Respondent answered “Yes” to DV-Q6 above.

## Independent Variables: Insurance instability

1. Experienced a change in insurance type, with no gap in coverage (INSURANCE CHURN)
2. Experienced a loss of insurance coverage (INSURANCE LOSS)

#### Relevant survey questions

*IV-Q1. Are you currently covered by any of the following types of health insurance?*

*(check all that apply)*

1. Insurance through current or former employer or union (by you or another family member). This would include COBRA coverage
2. Insurance purchased directly from an insurance company (by you or another family member). This would include coverage purchased through an exchange or marketplace, such as [state-specific examples] or Healthcare.gov
3. Medicare, for people 65 and older or people with certain disabilities
4. Medicaid ([state-specific examples]) or any kind of non-military government-sponsored plan based on income or disability, including coverage your clinic helped you obtain
5. TRICARE or other military health care, including VA health care
6. Indian Health Service
7. Iowa Family Planning Program (formerly Iowa Family Planning Network or IFPN)
8. Any other type of health insurance or health coverage plan, please specify any other plans:____________________
9. I do not currently have any health insurance or health coverage plan

IV-Q2: You just indicated that you have no insurance. This means you are not covered by any health care plan such as [state specific examples], or any other state program. Is that correct?

- - Yes
  - No, I do have health insurance, Specify type:_________________
  - Prefer not to answer

*Note: This question was added to Arizona, New Jersey, and Wisconsin surveys in waves 1 and 2. It was added to Iowa surveys in waves 2 and 3.*

IV-Q3: For how many of the past 12 months were you WITHOUT health insurance? (select one)

1. Less than 1 month
2. 1 to 3 months
3. 4 to 6 months
4. 7 to 12 months
5. Prefer not to answer

#### Variable construction:

- Respondent is considered to have experienced **INSURANCE CHURN** if they experienced a change in insurance type with no gap in coverage between two time points (i.e., response to IV-Q1 differs between time points).
- Respondent is considered to have experienced **INSURANCE LOSS** at follow-up if they were insured (i.e., selected a-h to IV-Q1 or “No, I do have health insurance” to IV-Q2) at a previous time point and uninsured (i.e., selected option i to IV-Q1 and/or “yes” to IV-Q2) at follow-up. Baseline respondents are marked as having lost insurance if they reported that, within the past twelve months, they were without health insurance for 6 months or less (i.e., selected option a, b, or c on IV-Q3). Given the categorical nature of IV-Q3, we were unable to distinguish between respondents who were without health insurance for 7-11 months from those who were continuously uninsured and thus respondents who reported 7-12 months of uninsurance in the preceding year (i.e., selected option d on IV-Q3) were not considered to have lost insurance, but as being continuously uninsured and thus not included in our analysis.

## Other covariates

### State

The Surveys of Women used in this analysis were fielded in Arizona, Iowa, New Jersey, and Wisconsin. These were separate fielding efforts producing separate datasets; we appended these datasets into one, maintaining state of residence information.

### Respondent age

CV-Q1: What year were you born?

____ year

Prefer not to answer

CV-Q2: [PROBE] What if I give you some categories?

1. Under 18
2. 18-19
3. 20-24
4. 25-29
5. 30-34
6. 35-39
7. 40-44
8. 45 or older
9. Don’t know
10. Prefer not to answer

#### Variable construction:

Respondents were grouped into three age categories based on their responses to the above questions: 18-25, 26-34, and 35+. The cut-off between 25 and 26 was chosen strategically to align with the age after which individuals are no longer eligible for coverage under their parent’s insurance.

### Educational attainment

CV-Q3. What is the highest degree or level of school you have completed?

1. Less than high school degree
2. High school graduate, GED or alternative credential
3. Some college or Associate’s degree
4. College graduate or more
5. Prefer not to answer

#### Variable construction

Respondents were grouped into three education categories based on their responses to the above question: High school or less, some college or Associate’s, and Bachelor’s or higher.

We then created dummy variables for each category in our hybrid logistic regression models.

### Employment

CV-Q4. Are you currently…? Check the response that best describes your status in terms of work/school.

1. Employed for wages
2. Self-employed
3. A student
4. Out of work for 1 year or more
5. Out of work for less than 1 year
6. A homemaker
7. Retired
8. Unable to work
9. Prefer not to answer

#### Variable construction

Respondents were grouped into two categories based on their responses to the above question: Employed or not employed.

### LGBTQ+ identity/orientation

CV-Q5. What sex were you assigned at birth, on your original birth certificate?

1. Female
2. Male
3. Other
4. Prefer not to answer

CV-Q6. How do you describe yourself? Check all that apply.

1. Woman
2. Man
3. Transgender
4. [Do not identify as male, female, or transgender [OR] Gender expansive]*
5. Something else, Specify: ____
6. Don’t know
7. Prefer not to answer

* Arizona, New Jersey, and Wisconsin baseline questionnaires included “gender expansive” as a response option, while all other states and waves included “do not identify as female, male, or transgender” in place of this response option.

CV-Q7. Which of the following best represents how you think about yourself? More than one response is possible.

1. Lesbian or gay
2. Straight, that is, not lesbian or gay
3. Bisexual
4. Pansexual
5. Queer*
6. Something else, Specify: ____
7. Don’t know
8. Prefer not to answer

* Follow-up surveys across all states and Iowa baseline questionnaires did not include “Queer” as a response option. New Jersey, Wisconsin, and Arizona baseline questionnaires did include “Queer” as a response option.

#### Variable construction:

We created a binary variable based on responses to the above three questions to categorize respondents as either “Cisgender, heterosexual” or “LGBTQ+.” Respondents reporting any sexuality other than “straight” are grouped into the “LGBTQ+” category, as well as those reporting trans, nonbinary, or other gender identity, and respondents whose assigned sex at birth does not match their gender identity. Only respondents reported both “straight” as their sexuality *and* whose gender identity aligns with their sex assigned at birth (e.g., assigned female at birth and reported “woman” as their gender identity) were coded as being “cisgender, heterosexual.” While we understand that gender and sexuality can both be fluid, for analytic and interpretive simplicity, we treat this as a time-invariant characteristic by using only the baseline response for each respondent.

### Marital status

CV-Q8. What is your current marital status?

1. Married
2. Separated
3. Widowed
4. Divorced
5. Never married
6. Prefer not to answer

CV-Q9. Do you currently have a main romantic partner, a spouse, a boyfriend or girlfriend, or someone you are more serious about than other people?

1. Yes
2. No
3. Prefer not to answer

CV-Q10. Do you currently live with a spouse or romantic partner?

1. Yes
2. No
3. Prefer not to answer

#### Variable construction

Marital status is constructed as a binary variable (married/unmarried) incorporating the below questions. Respondents reporting “married” are categorized as such, while all other respondents, whether in a current relationship, living with a partner, or not, are categorized as unmarried.

### Race/ethnicity:

CV-Q11. Do you consider yourself Hispanic, Latina, or Latinx origin?

1. Yes
2. No
3. Prefer not to answer

CV-Q12. Which one or more of the following best describes your race? Please check all that apply.

1. Black or African American
2. White
3. Asian or Asian American
4. Native American, Alaska Native, or American Indian
5. Native Hawaiian or Pacific Islander
6. Other, please specify:
7. Prefer not to answer

#### Variable construction

Respondents are categorized into a binary variable as either non-Hispanic white or Black, Indigenous, or other person of color (BIPOC), a label we use to refer to all non-white and non-Hispanic respondents in our sample. This includes non-Hispanic Black or African American, Asian or Asian American, Native American, Alaska Native, or American Indian, and Native Hawaiian or Pacific Islander respondents, as well as multiracial respondents and Hispanic respondents of all races including white. The authors would have preferred to maintain discrete racial and ethnic categories to explore any differential outcomes by race/ethnicity on a more granular level, but small cell sizes, particularly among our foreign-born population, made a binary variable more usable. We then created dummy variables for each category in our hybrid logistic regression models.

### Nativity

CV-Q13. Where were you born?

1. United States (including its territories: Puerto Rico, Guam, etc.)
2. Outside of the United States
3. Prefer not to answer

### Desire to Avoid Pregnancy (DAP):

We code DAP scores according to the recommendations of the team that created the scale at the University of California San Francisco: raw item scores are divided by 14 to obtain an average score regarding pregnancy preferences ranging from 0-4, where higher scores reflect a higher desire to avoid pregnancy. Further information on this scale is cited elsewhere:

Hall, Jennifer Anne, Geraldine Barrett, Judith M Stephenson, Natalie Lois Edelman, and Corinne Rocca. “Desire to Avoid Pregnancy Scale: Clinical Considerations and Comparison with Other Questions about Pregnancy Preferences.” *BMJ Sexual & Reproductive Health* 49, no. 3 (July 2023): 167–75. <https://doi.org/10.1136/bmjsrh-2022-201750>.

**CV-Q14. The following statements relate to your thoughts and feelings about *the idea of becoming PREGNANT in the next 3 months*. Even if you do not think you can become pregnant for partner or physical reasons, please imagine how you would feel about becoming pregnant.**

| Item | 01. Strongly Agree | 02. Agree | 03. Neither Agree nor Disagree | 04. Disagree | 05. Strongly Disagree | 99. Prefer Not to Answer |
| --- | --- | --- | --- | --- | --- | --- |
| I wouldn’t mind it if I became pregnant in the next 3 months. | □ | □ | □ | □ | □ | □ |
| It would be a good thing for me if I became pregnant in the next 3 months. | □ | □ | □ | □ | □ | □ |
| Thinking about becoming pregnant in the next 3 months makes me feel unhappy. | □ | □ | □ | □ | □ | □ |
| Thinking about becoming pregnant in the next 3 months makes me feel excited. | □ | □ | □ | □ | □ | □ |
| Becoming pregnant in the next 3 months would bring me closer to my main partner.  *(By main partner, we mean* ***the romantic partner that is the most serious to you****. If you don’t have a romantic partner, please think about the person with whom you were last sexual.)* | □ | □ | □ | □ | □ | □ |

**CV-Q15. [DAP2] The following statements relate to your thoughts and feelings about *the idea of having a BABY in the next year.* Even if you do not think you can have a baby for partner or physical reasons, please imagine how you would feel about having a baby.**

| Item | 01. Strongly Agree | 02. Agree | 03. Neither Agree nor Disagree | 04. Disagree | 05. Strongly Disagree | 99. Prefer Not to Answer |
| --- | --- | --- | --- | --- | --- | --- |
| I want to have a baby within the next year. | □ | □ | □ | □ | □ | □ |
| If I had a baby in the next year, it would be bad for my life. | □ | □ | □ | □ | □ | □ |
| It would be a positive addition to my life to have a baby in the next year. | □ | □ | □ | □ | □ | □ |
| It would be the end of the world for me to have a baby in the next year. | □ | □ | □ | □ | □ | □ |
| Thinking about having a baby within the next year makes me smile. | □ | □ | □ | □ | □ | □ |
| Thinking about having a baby within the next year makes me feel stressed out. | □ | □ | □ | □ | □ | □ |
| I would feel a loss of freedom if I had a baby in the next year. | □ | □ | □ | □ | □ | □ |
| If I had a baby in the next year, it would be hard for me to manage raising the child. | □ | □ | □ | □ | □ | □ |
| I would worry that having a baby in the next year would make it harder for me to achieve other things in my life. | □ | □ | □ | □ | □ | □ |

# Other methodological notes

In the Iowa baseline and follow-up 1 surveys, respondents who affirmed that they had had a tubal ligation or another operation (such as hysterectomy) that made them unable to get pregnant skipped a number of items including questions about their desire to avoid pregnancy (DAP). In all other surveys, items relevant to this analysis, including the DAP questions, were asked of all respondents. We have therefore excluded respondents whose sole contraceptive method was tubal ligation.

We note the use of the term “women” in the name of the survey from which our data are drawn but use the term “people” or “respondents” when describing the respondents in our analytic sample, as our sample includes people assigned female at birth who self-identified as "female”, “transgender”, “gender expansive”, or something else. Only respondents reporting “male” sex were excluded.
